# Women's Socioeconomic Status and Violence Against Women: The Case of Turkey

**DOI:** 10.1177/10778012261440227

**Published:** 2026-04-21

**Authors:** Elif Şeyban, Francesca Zanasi, Marco Albertini

**Affiliations:** 1 Department of Political and Social Sciences, 120694 University of Bologna, Bologna, Italy

**Keywords:** educational level, employment status, intimate partner violence, Turkey, amelioration, backlash

## Abstract

This study analyses the relationship between women's socio-economic status and intimate partner violence in Turkey using data from the Turkish National Research on Domestic Violence on 6,488 women. It explores if and how women's educational level and employment status influence the risk of physical, sexual, emotional, and economic violence, testing both the amelioration, which argues that women's empowerment reduces violence, and the backlash hypothesis, which suggests that men may respond to women's rising status with violence. Findings indicate that education is protective against intimate partner violence, supporting the amelioration hypothesis, while employed women and those more educated than their partners face higher risks, consistent with the backlash hypothesis. The study highlights the importance of adopting a multifaceted approach to women's empowerment.

## Introduction

Women worldwide face a heightened risk of gender-based violence compared to men, regardless of factors such as country, ethnicity, religion, or economic and social status (HUIPS, 2015). “Violence against women” (VAW) emerged as a critical social issue in the late 1960s and gained further attention with the second-wave feminist movement. This led to extensive research, activism, legal reforms, and the establishment of an international definition. The United Nations’ *Declaration on the Elimination of Violence Against Women*, adopted by the General Assembly in 1993, defines VAW as any gender-based act or threat that causes physical, sexual, psychological pain, or economic harm to women in both public and private spheres (Altınay & Arat, 2007; Kilpatrick, 2004; UN, 1993).

Estimates indicate that about one in three women worldwide have been subjected to either physical and/or sexual violence in their lifetime (WHO, 2021). According to the European Union Agency for Fundamental Rights (FRA, 2014), 43% of women in the EU report experiencing emotional violence, and 32% report being denied access to money by an intimate partner, further emphasizing the widespread nature of both emotional and economic abuse alongside other forms of violence.

VAW is linked to a wide range of health consequences, including physical injuries, mental health issues such as trauma, depression, and anxiety (Krug et al., 2002). Thus, VAW also imposes high costs on healthcare systems and society, weakening social cohesion by harming half of the population and perpetuating cycles of violence (Tjaden & Thoennes, 2000; Ulucan & Yüksel, 2021).

Feminist theories have put forward two different hypotheses about changing women's socio-economic status (SES) and the experience of violence: the amelioration hypothesis suggests that improving women's status reduces VAW (Martin et al., 2006; Whaley, 2001), while the backlash hypothesis posits that threatened male dominance may lead to increased violence (Chon, 2013; Martin et al., 2006). While power imbalances between genders contribute to VAW, the relationship between women's status and violence remains complex. This study explores these contrasting perspectives to gain a deeper understanding of VAW.

This research investigates how women's SES—measured through education and employment—shapes their likelihood of experiencing intimate partner violence (IPV), encompassing physical, sexual, emotional, and economic violence. To address this relationship, the study engages with both the amelioration and backlash hypotheses, drawing on data from the Turkish National Research on Domestic Violence Against Women 2014. In doing so, it situates the Turkish case within broader theoretical debates, while also contributing to the scientific understanding of IPV and to policy discussions on targeted interventions.

The paper is organized as follows. The next two sections provide the theoretical background on gender inequality and violence by reviewing key hypotheses and summarizing macro- and micro-level findings on VAW, which guide the formulation of the research hypotheses. This is followed by a discussion of the Turkish context, highlighting its relevance for the study. The section on “Analytic Strategy” presents the research hypotheses in relation to Turkey's gender dynamics, while the methodology section outlines the data and empirical approach. The results section reports findings from logistic regressions on physical, sexual, emotional, and economic violence. The paper concludes with a discussion and a summary of the main findings.

### Women's Status and Violence: Two Competing Perspectives

Gender inequality influences all areas of life—economic, social, legal, political, and familial—with violence representing one of the most severe manifestations, where perpetrators assert dominance over victims (Bulsari, 2021). Gender norms, which shape societal roles and behaviors, often create power imbalances that privilege men over women, granting men greater control and decision-making power. This disparity can lead to violence, particularly when men perceive threats to their masculinity (Anderson, 1997). Gender inequality is both ideological and structural; ideologically, it is rooted in societal beliefs and values about women's roles, while structurally, it is reflected in women's limited access to resources and societal positions (Dobash & Dobash, 1979; Yodanis, 2004). Discrimination in education, healthcare, and employment further exacerbates these disparities (Bulsari, 2021; UNDP, 2016). Research suggests that increasing gender equality at the societal level narrows the power gap between men and women, reducing the risk of VAW (Whaley et al., 2013; LeSuer, 2022). However, achieving gender equality requires, at the micro individual level, empowering women, especially through education and economic opportunities, which are crucial for altering power dynamics. While some argue that advancing gender equality could provoke a backlash, as men may perceive threats to their power, potentially increasing VAW (Bhardwaj & Apel, 2020; Lauritsen & Heimer, 2008). VAW is predicted to persist in societies with high gender inequality, where women's roles are heavily restricted (Whaley et al., 2013). Empowering women is essential in challenging these structural inequalities and reducing violence.

This complexity is reflected in two competing perspectives: the amelioration and backlash hypotheses. The amelioration hypothesis, grounded in feminist theory, argues that VAW stems from patriarchy and male dominance. By enhancing women's SES and promoting gender equality, it is believed that victimization—whether sexual, physical, emotional, or economic—will decrease as women gain better access to education, employment, income, and legal assistance (Dobash & Dobash, 1979; Vieraitis et al., 2007). At the micro level, this view is echoed in the Household Bargaining Theory, which suggests that improving women's status expands their external options and increases their bargaining power in relationships, thereby reducing their vulnerability to violence (Aizer, 2010; Farmer & Tientfaler, 1996; Stevenson & Wolfers, 2006).

In contrast, the backlash hypothesis, rooted in radical feminist literature, suggests a paradoxical effect where increased gender equality might actually escalate violence. As women make gains in traditionally male-dominated arenas, men may react with a “backlash,” employing social control strategies to maintain their relative power (Lauritsen & Heimer, 2008; Whaley et al., 2013). For instance, a wife's growing independence, marked by improved SES, may challenge cultural norms of male dominance, leading to violence as a means for men to reassert authority (Macmillan & Gartner, 1999). Similarly, as women's employment opportunities enhance their bargaining power, this shift can incite resistance or aggression from partners adhering to traditional gender roles (Field et al., 2021). These conflicting hypotheses highlight the nuanced interplay between gender equality and VAW, suggesting that while progress toward equality holds promise for reducing violence, it may also provoke resistance from those invested in maintaining traditional power structures.

## Literature Review

The relationship between women's status and VAW is complex, shaped by both macro-level societal factors and micro-level individual dynamics. Research examining this relationship has produced mixed findings, particularly regarding how factors such as education, labor market participation, and income equality influence the likelihood of VAW. Understanding these dynamics requires integrating both macro-level studies that explore societal trends and micro-level studies that focus on individual and family-level factors.

### Macro-Level Factors Affecting VAW

The ameliorative and backlash hypotheses regarding women's status have been explored through macro-level studies on education, labor force participation, and income (Wall, 2014), with mixed results regarding their impact on VAW. Whaley (2001) found that women's educational gains in U.S. cities were initially linked to higher rape rates, but shifts in the education gap between 1970 and 1990 contributed to a reduction in rape. In contrast, Eschholz and Vieraitis (2004) found that higher educational attainment exacerbates rape rates. Reckdenwald and Parker (2008) showed that gender educational inequalities increased women's homicide offending, while DeWees and Parker (2003) found a negative relationship with female homicide victimization. Jewkes (2002) attributed these conflicting results to the idea that while education and income may empower women, their effects are contingent upon cultural values and societal needs.

Regarding labor force participation, Austin and Kim (2000) and Ellis and Beattie (1983) found no significant link, while Eschholz and Vieraitis (2004) and Whaley (2001) suggested that greater inequality in labor force participation is associated with lower sexual violence. Martin et al. (2006) found that higher women's labor market participation is related to lower rape rates. In terms of income equality, Ellis and Beattie (1983) and Eschholz and Vieraitis (2004) showed no significant relationship, while Bailey (1999), Whaley (2001), Martin et al. (2006), and Reckdenwald and Parker (2008) supported the ameliorative hypothesis. Conversely, Pridemore and Freilich (2005), Baron and Straus (1987), and Peterson and Bailey (1992) reported a backlash effect on VAW. Austin and Kim (2000) suggested that increased vulnerability to sexual violence may result from women's increased activities outside the home as they progress toward equality.

Yodanis (2004), analyzing data from 27 countries, found that women's educational and occupational status was negatively correlated with sexual violence, but the relationship with physical violence was inconsistent. Yodanis also observed that more developed countries exhibit lower levels of violence, due to reduced traditional gender roles, while developing countries with dominant traditional gender roles tend to have higher levels of violence (Yodanis, 2004; Yüksel, 2021).

This literature review highlights the complex nature and mixed empirical evidence on the relationship between gender equality and VAW at the macro level. The conflicting results can likely be attributed to differences in data sources, methodologies, and the socio-cultural context of the countries studied. For our paper, we aim to advance this literature by focusing on micro-level factors—specifically, women's SES—and their direct relationship with IPV in Turkey. By using more localized data, we can explore the nuances of this relationship in a specific cultural and societal context, providing insights that are often lost in broader macro-level studies.

### Micro-Level Factors Affecting VAW

Previous studies have extensively studied the micro-level processes underlying the relationship between women's labor market participation, education level, income autonomy, and IPV, also studying this relationship in countries at different stages of social and economic development.

Chin (2012) showed that working women in India are more likely to experience physical violence, thus supporting the Backlash Theory. Paul (2016) also found that working women in India, particularly those with higher earnings or income control, face an increased likelihood of IPV in terms of physical as well as emotional abuse. In Jordan, Lenze and Klasen (2017) found that women in paid employment are more likely to experience emotional and physical IPV. However, their analysis also indicates that employment may offer a protective effect against sexual violence, although this evidence is weak. In contrast, studies in the U.S. by Aizer (2010) and Stevenson and Wolfers (2006) align with the “Household Bargaining Model,” indicating that women's increased labor force participation and improved market conditions empower women, helping to reduce gender wage gaps. This economic empowerment lowers the likelihood of women experiencing IPV, as it enhances their bargaining power within relationships. In Turkey, while some studies found no statistically significant relationship between employment and IPV (Büyükyılmaz & Demir, 2016), others identified a negative association, particularly for women in formal employment (Dasre et al., 2017).

The impact of education is more consistently negative across studies, with evidence from India (Ackerson et al., 2008; Eswaran & Malhorta, 2011), Turkey (Dasre et al., 2017; Ediz & Altan, 2017; Ergin et al., 2005; Kocacık & Doğan, 2006), Lebanon (Ustaa et al., 2007), and China (Tang & Lai, 2008) showing that higher education levels are associated with lower exposure to IPV. This suggests that education—more than employment—acts as a protective and ameliorative factor, potentially equipping women with knowledge, skills, and resources to navigate challenging situations and assert their rights.

Since educational level and employment status are key measures of economic independence, enhancing earning potential and financial security, it is crucial to consider how these factors relate to violence. Studies show mixed results: economic independence, measured through wage equality (Aizer, 2010), property ownership (Dasre et al., 2017), and income autonomy (Ulucan & Yüksel, 2021), often correlates with reduced violence, suggesting an amelioration effect (Basu & Famoye, 2004; Kocacık & Doğan, 2006). However, some studies indicate a potential male backlash, with a positive association between women having higher income than their partners, complete income control, and increased violence (Paul, 2016), reflecting tensions as traditional gender roles are challenged.

In addition to the sociological and demographic perspectives discussed above, a growing body of economic research has leveraged causal identification strategies, often through quasi-experimental or experimental designs, to examine the relationship between women's SES and IPV. In Turkey, Erten and Keskin (2018) exploited the 1997 compulsory schooling reform as a natural experiment, finding that while higher female education improved women's labor market outcomes, it also increased reports of psychological violence and financial control in rural areas, with no significant effects on physical or sexual violence. In Mexico, Bobonis et al. (2013) evaluated a randomized conditional cash transfer program and showed that transfers targeted to women reduced physical abuse by about 40 percent but increased threats and emotional coercion. In Bangladesh, Heath (2014) used quasi-experimental variation from women's labor market participation and found that employment is associated with higher IPV risk for women with low bargaining power, while no such risk is evident for women with stronger status, illustrating the conditional nature of backlash dynamics. Together, these studies demonstrate that economic reforms and shocks can shape IPV in heterogeneous ways, with some policies reducing certain forms of violence while exacerbating others, thereby providing rigorous causal evidence on both the amelioration and backlash hypotheses. By contrast, the present study adopts a descriptive approach based on nationally representative survey data, which, although not causal in design, enables a nuanced analysis of multiple forms of violence within the Turkish case.

Other factors influencing VAW include age, with studies by Ediz and Altan (2017) suggesting a higher risk for women aged 30–40, while Dasre et al. (2017) found a decreased risk with increasing age. Partner characteristics such as low education, unemployment, substance use, and the presence of children also contribute to the risk of violence (Alkan & Tekmanlı, 2021; Büyükyılmaz & Demir, 2016; Dasre et al., 2017). These studies highlight the complex relationship between women's SES and IPV, emphasizing the need for a multifaceted approach that considers education, employment, income autonomy, and partner dynamics.

## Measuring VAW

In the existing literature, IPV is operationalized in various ways, often grouped under a general IPV or domestic violence umbrella (Ackerson et al., 2008; Eswaran & Malhorta, 2011) or assessed through distinct forms of violence. Some studies create a composite measure of IPV, categorizing any instance of physical, emotional, sexual, or economic violence as indicative of IPV. For example, Dasre et al. (2017) analyzed physical and sexual violence, while Ustaa et al. (2007) considered psychological, emotional, physical, and economic violence, defining IPV as present if any of these types is reported. This approach provides a broad overview but can obscure the specific impacts of each type of violence. Conversely, other researchers measure these forms of violence separately, allowing for a more nuanced analysis of how different socio-economic factors influence each type, as seen in the work of Ergin et al. (2005) and Tang and Lai (2008), who assessed physical, psychological, and sexual violence distinctly. Paul (2016) focused on both emotional and physical violence, while Chin (2012) specifically examined physical spousal violence. Lenze and Klasen (2017) bridged these approaches by initially combining emotional, physical, and sexual violence into a single index for regression analysis and then performing robustness checks where each type of violence is analyzed separately across a lifetime.

Additionally, the timeframe of violence assessment varies among studies, with some examining IPV over a lifetime (Ackerson et al., 2008; Büyükyılmaz & Demir, 2016; Kocacık & Doğan, 2006; Lenze & Klasen, 2017; Tang & Lai, 2008) and others focusing on recent occurrences within the last year (Chin, 2012; Dasre et al., 2017; Paul, 2016). This temporal variation provides insights into both the immediate and long-term effects of socio-economic conditions on IPV prevalence and severity.

In the present paper, we employ separate measures for different forms of IPV, analyzing each type of violence individually within the last 12 months. This approach enables a comprehensive and detailed analysis of the unique contributions and prevalence of each form of violence, including physical, emotional, sexual, and economic. Focusing on recent experiences of violence, the paper aims to provide a dynamic and current understanding of IPV. This methodology is especially important for thoroughly investigating economic violence, a less frequently examined aspect, thereby filling a significant gap in the literature and contributing to the development of more targeted and effective intervention strategies.

## The Turkish Case

Turkey provides a compelling case study for examining IPV due to its unique socio-cultural and economic dynamics shaped by traditional gender roles and patriarchal structures. According to the 2021 Global Gender Gap Index, Turkey ranks 133rd out of 156 countries (World Economic Forum, 2021), reflecting significant gender disparities. Cultural, religious, and traditional expectations restrict women's opportunities in education, employment, and wage advancement (Kaya, 2009), with conservative gender norms, especially in rural areas, exacerbating these barriers (Dasre et al., 2017). Despite legal reforms aimed at promoting gender equality, progress has been slow, particularly in the areas of behavioral change and the effective implementation of laws, leaving women to face persistent challenges in overcoming gender disparities, particularly in education and employment (TDHS, 2014). Although female employment has gradually increased, particularly in urban service sectors, it remains one of the lowest among OECD countries, with a high rate of informal and unpaid labor (Çin, 2021; Demir, 2021). These structural challenges provide a critical context for understanding the prevalence of IPV in Turkey.

Regional disparities in women's labor market participation were striking in 2014. Female employment rates in western regions such as Thrace, Izmir, and the Aegean exceeded 30%–35%, whereas in Eastern and Southeastern Anatolia, they frequently fell below 20%, reaching as low as 15% in the Southeast (OECD, 2016). Educational inequalities mirror this divide: in Southeast Anatolia, the female net attendance ratio at the primary level was around 71%, compared to nearly 96% in the Aegean region (Gumus & Gumus, 2013). More broadly, only about 61% of females in eastern provinces have ever been to school, versus 85% in the west (Tan, 2007), and girls in the East have historically been 1.5–3 times less likely to complete junior high school than those in western, central, or northern Turkey (Caner et al., 2016). These disparities underscore the marked regional heterogeneity in women's socioeconomic opportunities: conservative gender and family norms, particularly prevalent in the East, reinforce patriarchal structures and limit women's autonomy, while Western provinces tend to exhibit more egalitarian values. Such differences are likely to shape both the prevalence of IPV and the ways in which women's SES interacts with violence, making it necessary to interpret empirical evidence with this heterogeneity in mind (Dasre et al., 2017).

## Analytic Strategy

This study investigates the relationship between women's SES—education and employment—and their experience of IPV (physical, sexual, emotional, and economic) in Turkey. Physical violence is the intentional use of physical force with the intention to harm the victim, the extreme of which could be death (Bulsari, 2021; Krantz & Garcia-Moreno, 2005). Sexual violence is defined as any sexual attempt to obtain a sexual act against a person's sexuality using coercion, by anyone, regardless of their relationship to the victim (Jewkes et al., 2002). Emotional violence includes behaviors such as verbal abuse, threats, emotional manipulation, and coercion, which are associated with long-term mental health consequences like depression and anxiety (Walker, 1979; Johnson & Ferraro, 2000; Tjaden & Thoennes, 2000). Economic violence, on the other hand, involves controlling or limiting access to financial resources, further entrenching gender inequality and dependence, and reducing a woman's autonomy within the relationship (Adams et. al., 2008 Postmus et al., 2012).

The mixed results reported in previous studies suggest that it remains an open question whether empowering women in the Turkish context could actually work in the direction of reducing or augmenting IPV, according to the amelioration and backlash perspectives, backbones of our approach. The present study is divided into three different sections.

Firstly, we explore the relationship between education and various forms of IPV. Education is not only a measure of knowledge, but also a factor that shapes values, improves the ability to recognize abuse, and strengthens the capacity to seek help. It expands women's access to resources, reducing their dependence on their husbands and encouraging them to challenge traditional gender roles. As a result, highly educated women may be less vulnerable to IPV. Research supports a negative correlation between education and IPV, with studies suggesting that higher educational attainment reduces the likelihood of women accepting violence or conforming to traditional roles (Dasre et al., 2017; Ediz & Altan, 2017; Ergin et al., 2005; Kocacık & Doğan, 2006). While education is expected to be closely linked with reductions in physical and sexual violence, its impact on emotional and economic violence may be particularly profound. Educated women, given their higher independence and enhanced ability to recognize abusive dynamics, may be better equipped to resist and reject emotional manipulation and economic control from their partners.

Secondly, we analyze the relationship between employment status (formal/regular and informal/irregular) and different forms of IPV. Employment can be an important measure of women's economic independence, self-actualization, and their ability to interact with the broader community outside the household. Given the gender inequality in Turkey regarding women's employment and drawing on previous studies (Chin, 2012; Eswaran & Malhorta, 2011; Lenze & Klasen, 2017; Paul, 2016), many women face discrimination, unequal pay, and difficulties securing formal employment due to patriarchal norms and insufficient legal and institutional support. These factors could influence women's experience of IPV. We expect that employment will generally be associated with lower rates of economic violence, as women who are no longer financially dependent on their partners may gain greater control over their finances. However, the relationship with emotional and other forms of violence may differ. For instance, employed women might face emotional backlash from partners who perceive their employment as a challenge to traditional gender roles. This section will explore these expectations in detail.

Lastly, our study explores the role of partner characteristics by adopting a dyadic perspective. On the one hand, it considers the fact that couples are often homogamous, meaning they have the same educational and/or employment level. At the analytical level, studying women's characteristics without those of their partners could hide a partially spurious effect: partners’ educational level and employment could actually be the drivers of IPV. On the other hand, the study of heterogamous couples—specifically where women have higher education or better employment status than their partners—offers a valuable test for the backlash hypothesis. By analyzing heterogamy, as Pandian (2024) does in the context of India, we can examine whether IPV is used by partners to re-establish traditional gender roles.

## Data and Methods

### Data

The Domestic Violence Against Women Survey, analyzed in this study, was conducted in 2014 by the Institute of Population Studies at Hacettepe University, with support from TURKSTAT and funding from the Turkish Ministry of Family and Social Policies. The decision to use data from 2014 was based on the fact that it represents the most recent survey on VAW conducted in Turkey at the time. The survey was carried out across Turkey, covering a sample of 11,247 households representing 12 statistical regions, including both urban and rural areas. Face-to-face interviews were conducted with 7,462 women aged 15 to 59. Each household contributed responses from only one woman. If multiple women within the eligible age range were present, one was selected randomly to prevent duplicate responses to the same questions within a household.

### Sample

Given that IPV constituted a significant proportion of overall VAW, the study sample was restricted to women who had ever been in a partnership and were aged 18 or older. The final dataset included 6,488 observations with complete data on the variables of interest.

### Variables

The dependent variables measured physical, sexual, emotional, and economic violence experienced by women from an intimate partner within the last 12 months. Each variable was coded as one if the woman had experienced that form of violence and zero if she had not. Physical violence included actions such as slapping, punching, kicking, burning, and using or threatening to use a gun or knife. Sexual violence encompassed experiences of rape, sexual assault, or sexual harassment by a partner in the past year. Emotional violence was assessed by asking whether, in the past 12 months, the respondent's partner had insulted, humiliated, intimidated, or threatened harm to her or her loved ones. Economic violence was determined based on whether the woman had been prevented from working despite wanting to, forced to quit her job, denied household financial support despite the partner's means, or deprived of her own income against her will.

The first independent variable related to women's educational attainment is categorized into three levels. The low level included women who had never attended school, attended but did not complete primary school, or completed primary school. The middle level included women who completed middle school or high school. The tertiary level included women who obtained an undergraduate, postgraduate, or doctoral degree.

The second independent variable pertained to women's employment status, classified into three categories. Regular or formal employment included employers, regularly waged workers, public sector employees, and self-employed professionals. Irregular or informal employment included seasonal or temporary workers, irregular self-employed individuals, and unpaid family workers. The not-employed category included inactive individuals, unemployed people, retirees, and students.

Key covariates included partner-related variables such as the partner's education level, classified into low, middle, and tertiary levels, and the partner's employment status, categorized as regular or formal employment, irregular or informal employment, or not working. Additionally, the models controlled for geographic residence, distinguishing between urban and rural areas, as well as age, number of children, and relationship status, which indicated whether the woman was currently in a relationship or not. To test for potential backlash effects, an additional variable was created to assess the couple's relative education. This variable indicated whether the partners had the same educational level, whether the woman had a higher level of education than her partner, or whether the partner had a higher level of education than the woman.

## Methods

**Figure 1.**
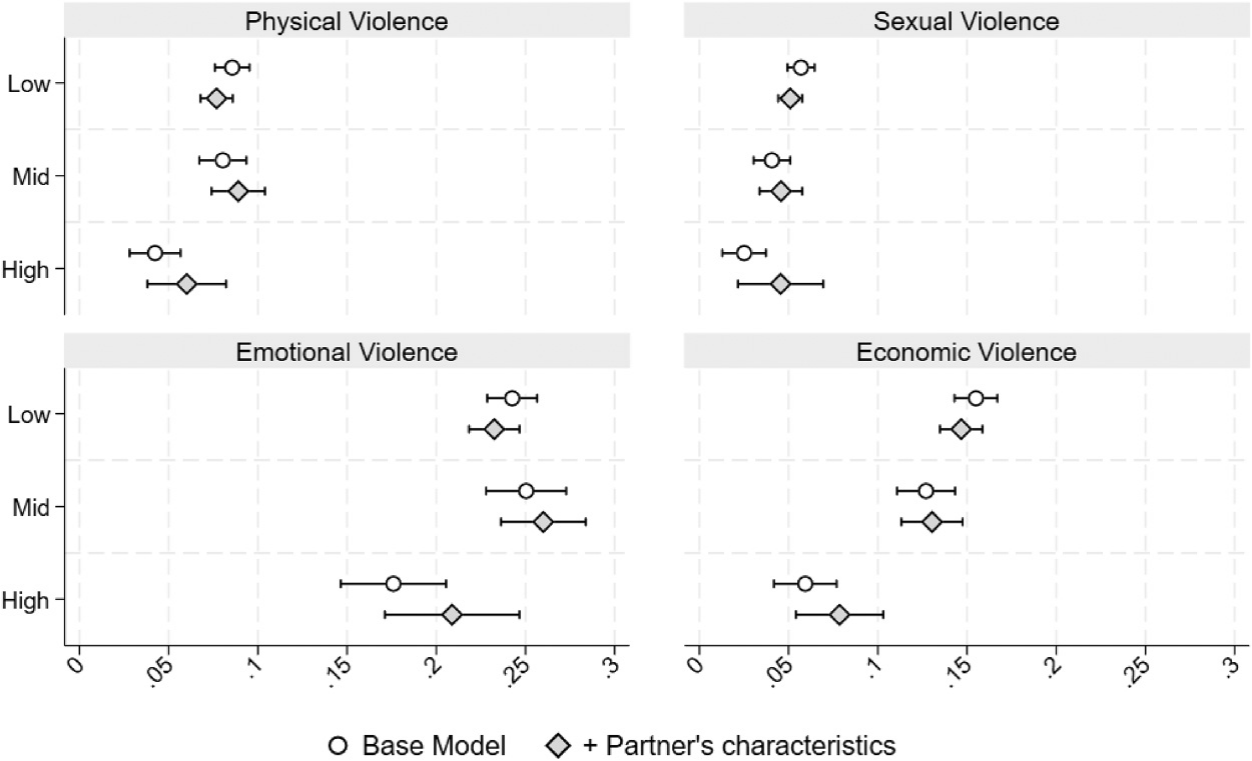
Predicted probabilities of experiencing physical, sexual, emotional, and economic violence, by a woman's level of education. Note: 95% CI. Results from logistic regression models. “Base Model” (white circles) includes woman's characteristics: age, urban/rural, region, number of children, employment status, and whether in a relationship; Model “+ Partner's Characteristics” (gray diamonds) adds controls for partner's education and employment status. *N *= 6,488.

**Figure 2.**
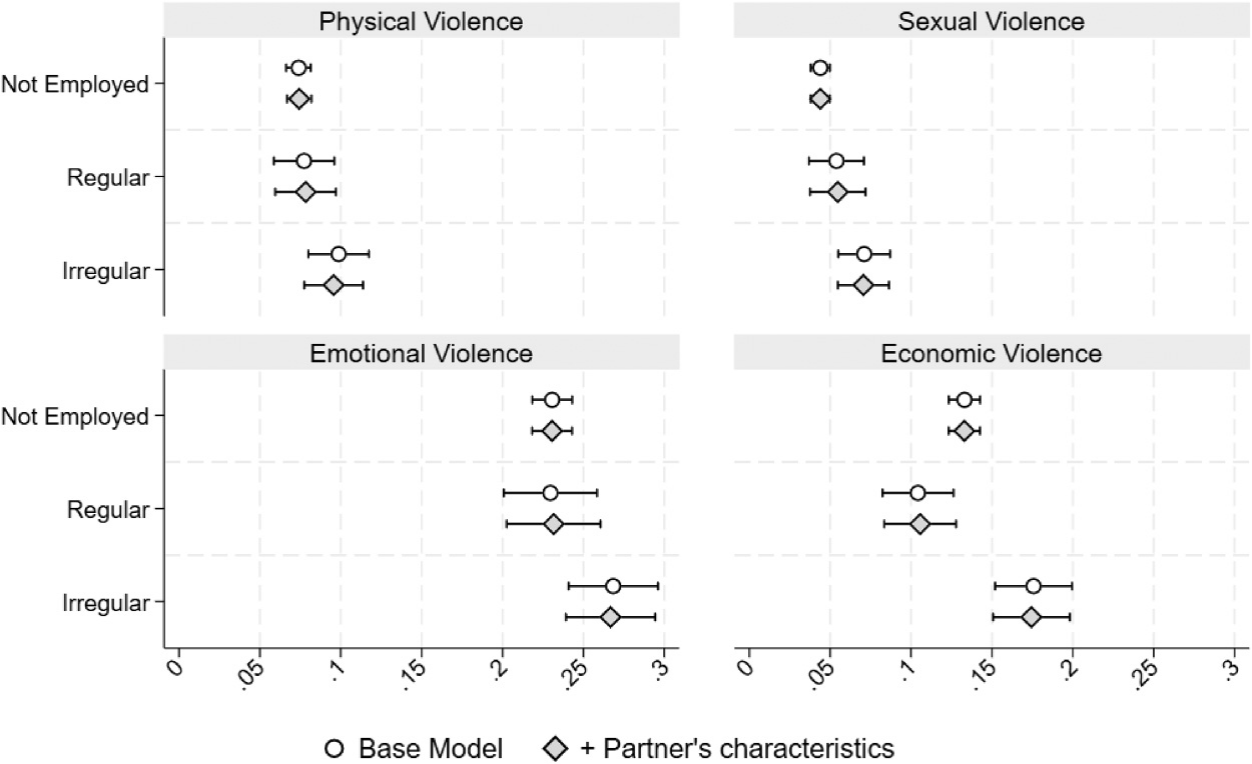
Predicted probabilities of experiencing physical, sexual, emotional, and economic violence by a woman's employment status. Note: 95% CI. Results from logistic regression models. “Base Model” (white circles) includes woman's characteristics: age, urban/rural, region, number of children, educational level, and whether in a relationship; Model “+ Partner's Characteristics” (gray diamonds) adds controls for partner's education and employment status. *N* = 6,488.

Given the dichotomous nature of the dependent variables, a series of logistic regression models was estimated. A stepwise approach was employed. The initial models incorporated two primary socioeconomic variables, education level and employment status, along with key socio-demographic controls, including age, number of children, relationship status, urban/rural, and region of residence. Partner-related characteristics were then added to the models (Figures 1 and 2).

**Figure 3.**
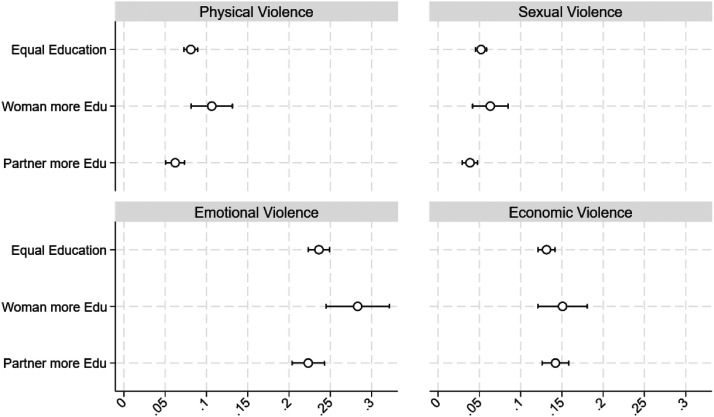
Predicted probabilities of experiencing physical, sexual, emotional, and economic violence, by a woman's and partner's education (relative measure). Note: 95% CI. Results from logistic regression models with all control variables included: age, urban/rural, region, number of children, employment status, whether in a relationship, partner's education, and employment status. A variable on the woman's and partner's relative education is added. *N* = 6,488.

**Figure A1.**
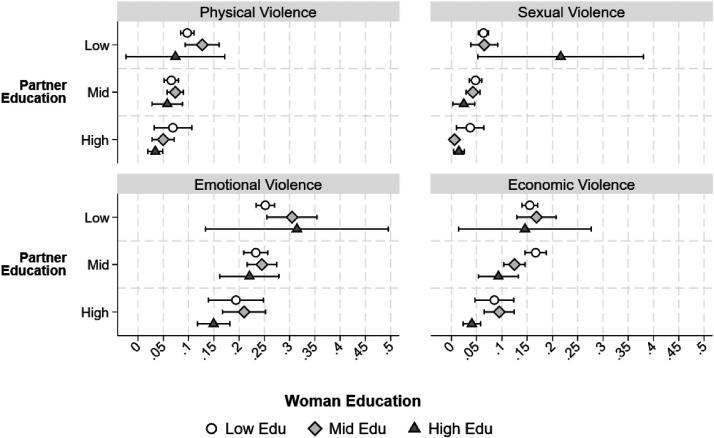
Predicted probabilities of experiencing physical, sexual, emotional, and economic violence, by women’s and partners’ education (absolute measure). Note: 95% CI. Results from logistic regression models with all control variables included age, urban/rural, region, number of children, educational level, employment status, whether in a relationship, partner's education, and employment status. Interaction terms were added between the woman's and partner's levels of education. *N* = 6,488.

Two additional models were estimated to analyze the interaction between women's and their partners’ education levels. In one model, the variable for women's education was replaced with the couple's relative education level (Figure 3). In another model, an interaction term between women's and their partners’ educational attainment was introduced to assess the absolute effect of education (Figure A1).

**Figure A2.**
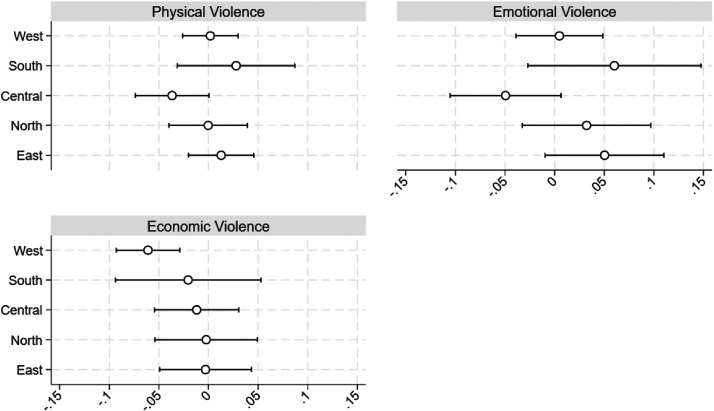
Difference in probability (average marginal effects (AMEs)) of experiencing physical, emotional, and economic violence, between women with high and low educational levels, by region of residence. Note: 95% CI. Results from logistic regression models with all control variables included age, urban/rural, region, number of children, educational level, employment status, whether in a relationship, partner's education, and employment status. Interaction terms were added between the woman's level of education and the region. Results from sexual violence were not estimable because of naught cell size in the combination highly educated # North region # experience of sexual violence = 1. *N* = 6,488.

Finally, the sensitivity analysis reported in Figure A2 explores regional heterogeneity: results from a logistic regression model adding an interaction term between women's education and region of residence are reported as average marginal effects. They show the difference in probability of experiencing physical, emotional, and economic violence between women with high and low educational levels, by region of residence. As no noteworthy differences are detected across regions in Turkey, the results are not commented on in the main text.

## Results

### Descriptive Results

Table 1 presents descriptive statistics on the variables used in the analysis, categorized by women's educational levels. The results indicated that women with higher education levels experienced lower rates of IPV across all forms (physical, sexual, emotional, and economic). Women with lower education levels were less likely to be employed compared to those with higher education (72% vs. 43%). Among highly educated women, 53% were in regular employment, compared to only 8% of low-educated women. A strong relationship emerged between women's and their partners’ educational and employment status. Specifically, 67% of low-educated women had low-educated partners, whereas 71% of highly educated women had highly educated partners. Similarly, low-educated women were more likely to have unemployed or informally employed partners, while highly educated women predominantly had regularly employed partners.

**Table 1.** Descriptive Statistics, by Women's Education: Column Percentages.

	Women's Education		
Variables	Low	Mid	High
Intimate partner violence			
Physical	8%	10%	5%
Sexual	6%	4%	2%
Emotional	23%	27%	19%
Economic	14%	15%	6%
Women’s employment			
Not employed	72%	71%	43%
Employed: Regular	8%	16%	53%
Employed: Irregular	20%	13%	4%
In a relationship: yes	93%	91%	84%
Age	41.14	32.68	32.79
Urban	61%	80%	92%
Region			
West	30%	34%	38%
South	7%	9%	9%
Central	20%	23%	25%
North	14%	15%	13%
East	29%	19%	15%
No. of children	2.86	1.60	0.99
Partner's education			
Low	67%	21%	3%
Mid	29%	56%	26%
High	5%	22%	71%
Partner's employment			
Not employed	22%	11%	13%
Employed: regular	58%	80%	84%
Employed: irregular	20%	9%	2%
Total	4,236	1,515	737

As far as Turkish regions are concerned, the distribution across regions is quite similar for each level of education.

### Women's Education and the Experience of IPV

The data reveal a clear and consistent relationship between women's education and the probability of experiencing violence, offering strong support for the amelioration hypothesis. Figure 1 shows that higher levels of education among women are generally associated with a lower probability of experiencing physical, sexual, emotional, and economic violence, supporting the amelioration hypothesis.

The probability of experiencing physical violence is approximately 8% for low-educated women and 4% for highly educated women. Similarly, the probability of sexual violence is 5% to 2%, respectively. For emotional violence, the probability ranges from 25% among low-educated women to 18% for highly educated women. Economic violence surely is the domain where women's education is more protective against partners’ violence: the risk is 16% for low-educated women, and 6% for highly educated women. Overall, the inclusion of partners’ characteristics (“+ Partners’ Characteristics” (gray diamonds)) only slightly contributes to explaining the educational gradient in IPV. However, the confidence intervals—especially between the mid and high education groups—tend to overlap, suggesting that not all observed differences are statistically significant.

### Women's Employment and the Experience of IPV

Figure 2 explores the role of employment status. The most exposed to partners’ violence, of any type, seem to be women with irregular employment contracts; to the contrary, scarce are the differences between women not in employment, and women holding regular contracts. For physical violence, women not in employment and with regular contracts have around 6% probability to experience violence, against the 10% of women with irregular contracts. Such numbers are 5% and 8% for sexual violence, and 22% and 27% for emotional violence. Economic violence represents an exception: while women with irregular contracts remain the most exposed (around 18%), not employed women have a statistically significantly higher probability of experiencing violence than women in regular employment (12% and 10%, respectively). The inclusion of partners’ characteristics (“+ Partners’ Characteristics” (gray diamonds)) does not account for explaining the employment differences in the experience of violence.

### IPV According to Educational Heterogamy

Figure 3 explores how relative educational attainment within couples relates to IPV. Generally, women who are more educated than their partners show higher predicted probabilities of experiencing all forms of violence. Focusing on statistically significant differences, for physical and sexual violence, there is a lower probability of experiencing IPV when the partner is more educated than when the woman is more educated (−0.04 and −0.02 p.p., respectively), or the partner has equal education (−0.02 and −0.01 p.p., respectively).

For emotional violence, when the partner has more education, the probability of experiencing IPV is 0.06 lower than when the woman has higher education. Also, when the woman has more education, the probability of experiencing IPV is 0.05 p.p. higher than when the partners have equal education. Overall, this pattern could be seen as aligning with the backlash hypothesis.

For economic violence, finally, there are no statistically significant differences in the probability of experiencing IPV according to partners’ education.

Figure A1 provides additional evidence on the interaction between women's and men's characteristics on the probability of experiencing violence, using an absolute measure of education. Despite very large confidence intervals, because of the low number of couples where the woman is more educated than the man, the results largely confirm those displayed in Figure 3.

## Discussion

In this study, we explored the relationship between women's socio-economic resources and the experience of IPV in Turkey. The findings indicate a discernible pattern where the experience of any form of violence—physical, sexual, emotional, and economic—is lower the higher the women's educational attainment. Education emerges as a pivotal factor fostering heightened awareness and the cultivation of personal skills, thereby empowering women to augment their capacity for self-protection against violence. These findings are consistent with prior research (Ackerson et al., 2008; Dasre et al., 2017; Eswaran & Malhorta, 2011; Ediz & Altan, 2017; Ergin et al., 2005; Kocacık & Doğan, 2006; Tang & Lai, 2008; Ustaa et al., 2007) and align with the amelioration hypothesis posited by feminist scholars in macro-level studies, highlighting the mitigating influence of increased educational attainment on the occurrence of IPV.

Regarding women's employment, the findings suggest that women who are employed with informal/irregular contracts are more likely to have experienced intimate violence compared to their non-employed and regularly employed counterparts. The phenomenon can be contextualized within the framework of the backlash hypothesis by supporting literature (Chin, 2012; Lenze & Klasen, 2017; Paul, 2016). In this context, the resistance towards a woman's engagement in employment can be seen as a manifestation of backlash against the perceived deviation from traditional gender norms. The desire of women to create their own freedom space and contribute to family income through work may be viewed as a threat to established gender roles, especially if such aspirations challenge the traditional notion of the male breadwinner. Consequently, any economic improvement in women can have the opposite effect on men due to the fear that the economic balances will change and cause IPV. Building on this understanding, the analysis clearly shows that formal employment offers protective benefits against all forms of IPV that are almost equivalent to those of unemployment, compared to informal or irregular employment. In our interpretation, women in formal employment settings benefit from superior employment security measures, supportive networks, and greater access to resources. This contributes to a lower vulnerability to physical, sexual, emotional, and economic violence. Particularly noteworthy is the pronounced protection formal employment provides against economic violence, underscoring the role of financial independence and bargaining power in sheltering women from IPV. This aligns with Aizer's (2010) “Household Bargaining Theory,” which posits that enhancements in labor market conditions improve a woman's bargaining power within domestic settings, thereby leading to a decrease in violence. The lower likelihood of experiencing IPV for women in formal employment emphasizes the critical need for policies that enhance the quality and stability of employment as a strategic approach to combat IPV.

The significant divergence in the social backgrounds of women engaged in formal and informal employment in Turkey highlights the distinct socio-economic contexts that shape women's experiences in the workforce. Women in formal employment often have a higher level of education and are likely to be driven to participate in the labor market not only by economic considerations but also by a pursuit of self-fulfillment, independent of their partners’ status. These women typically have partners with comparable educational backgrounds, underscoring a robust interplay between female education, the likelihood of securing regular employment in the formal sector, and being partnered with an educated and high-earning individual. Disentangling these relationships may not always yield meaningful distinctions, but collectively, these factors significantly contribute to a reduced risk of physical violence. This complex matrix of educational attainment, employment in formal sectors, and partner equivalence in educational and economic status serves to buffer women from the risks associated with IPV.

When exploring partners’ characteristics over women's characteristics, two interesting results emerged. On the one hand, we confirmed that women's characteristics are more important in explaining the experience of violence than their partners’; operationally, our results were not altered by the inclusion in the models of partners’ characteristics, suggesting that educational homogamy is not too important a part of the story. On the other hand, partners’ characteristics do matter when there is a power imbalance in the couple, signaled by a different level of education between the partners. Even though the differences were small in size, women who had more education than their partners were more exposed to the experience of intimate violence.

Overall, our findings show that while higher education can serve as a protective factor against violence (amelioration hypothesis), educational imbalances in favor of women can provoke backlash, resulting in higher violence risk (backlash hypothesis). These two explanations, amelioration and backlash, are not necessarily alternative but rather complementary, highlighting the dual influence of empowerment and resistance to shifting power dynamics.

This study has some limitations. Firstly, the cross-sectional design of the data precluded the establishment of clear causal relationships between women's status and their experience of violence, allowing only for the identification of associations. Anyway, we use a measure of IPV that refers to violence experienced in the last 12 months. This avoids major problems of reversed causality, e.g., women who have experienced violence may have been forced to leave school or withdraw from employment. Secondly, it employs outdated data 2014. To the best of our knowledge, the scarcity of up-to-date micro-level data on IPV is not unique to Turkey but is a widespread issue across Europe and beyond. Many national surveys on VAW, including those conducted by the European Union Agency for Fundamental Rights (FRA, 2014) and national studies in countries like Italy (ISTAT Violence against women survey 2014), are outdated, limiting the ability to capture recent, evolving patterns of IPV. This lack of recent data hinders the advancement of contemporary IPV research and the development of effective, evidence-based interventions.

Thirdly, although our analysis is framed around the amelioration and backlash hypotheses, the dataset does not include variables such as decision-making power, autonomy, or gender role attitudes that would allow us to test the underlying mechanisms directly. Decision-making power refers to the extent to which women are able to influence choices regarding issues such as household income or health care, which could strengthen their bargaining position and reduce exposure to IPV. Autonomy reflects the ability of women to move, work, or allocate resources independently of their partners, thereby decreasing dependence and vulnerability. Gender role attitudes concern beliefs about appropriate roles for men and women; for example, women with higher education may adopt more egalitarian perspectives, making them less likely to accept violence as legitimate. These pathways illustrate how SES may translate into differences in IPV risk. Without such measures, our interpretation of amelioration and backlash remains suggestive, and future research using richer data will be necessary to test these mechanisms explicitly.

Finally, reliance on self-report data exposes one to the possibility of underreporting or misreporting experiences of violence, given the sensitive nature of the topic. To mitigate this risk, the survey followed internationally acknowledged ethical and safety guidelines, including the use of a neutral study name, obtaining informed consent, interviewing only one woman per household in private settings, and ensuring strict confidentiality. Moreover, the questionnaire avoided stigmatizing terms such as “abuse” or “rape” and instead asked about specific acts of violence in a non-judgmental manner, which has been shown to increase reporting accuracy. These measures aimed to create a safe environment for disclosure and to enhance the reliability of responses, although some degree of underreporting cannot be entirely excluded (for a deeper discussion, please see Hacettepe University Institute of Population Studies & General Directorate on the Status of Women, 2015).

These limitations suggest some caution in the interpretation of the findings and highlight the urgent need for more frequent and comprehensive data collection efforts to better understand and address the complex dynamics of IPV globally.

The present study has highlighted that the relationship between women's status and VAW in Turkey cannot be explained by using only one theory or in one direction. Instead, there is a mix of mechanisms that explain how the likelihood of experiencing violence is affected by women's status. The analysis emphasizes the importance of adopting nuanced approaches and comprehensive strategies to address VAW. Understanding the multiple and diverse underlying mechanisms is crucial for devising effective policies and initiatives that genuinely empower women and safeguard their safety and well-being. Furthermore, addressing the root causes of violence, such as patriarchal attitudes and norms, requires a concerted effort to transform societal mindsets and promote gender equality.
